# Survival and life expectancy inequality by gender in Thai provinces: Trends from 2015 to 2023

**DOI:** 10.1371/journal.pone.0348587

**Published:** 2026-05-13

**Authors:** Sergei Scherbov, Wiraporn Pothisiri, Orawan Prasitsiriphon, Dalkhat M. Ediev, Warren Sanderson

**Affiliations:** 1 International Institute for Applied Systems Analysis, Laxenburg, Austria; 2 College of Population Studies, Chulalongkorn University, Pathumwan, Bangkok, Thailand; 3 North-Caucasian State Academy, Cherkessk, Russia; 4 Lomonosov Moscow State University, Moscow, Russia; 5 Department of Economics, Stony Brook University, Stony Brook, New York, United States of America; Freelance Consultant, Myanmar, MYANMAR

## Abstract

**Background:**

Gender disparities in survival and life expectancy are indicative of broader health and socio-economic inequalities. This study examines temporal trends and subnational variations in gendered survival outcomes across Thailand’s 77 provinces between 2015 and 2023, emphasizing the impacts of the COVID-19 pandemic and environmental factors. For the first time, comprehensive provincial life tables have been produced for all Thai provinces for this period.

**Methods:**

Using civil registration data, life tables were constructed, from which life expectancy at birth ($e_{\text{0}}$), at age 65 ($e_{\text{6}\text{5}}$), and survival probabilities between ages 20 and 65 were derived. Generalized additive models (GAM) with Poisson likelihood were employed to estimate mortality rates and analyze temporal trends and regional disparities. All analyses were conducted separately by sex. National-level estimates were computed as population-weighted aggregates of provincial estimates, from which life table indicators were derived consistently. Uncertainty intervals were estimated for key indicators.

**Results:**

From 2015 to 2023, the national gender gap in life expectancy at birth widened from 7.0 years to a peak of 7.7 years in 2021, primarily driven by increased male mortality during the COVID-19 pandemic. Provincial variations were substantial, with gender gaps ranging from 5.7 to 9.5 years. PM2.5 exposure measured in 2024 showed a moderate to strong negative correlation with life expectancy, highlighting significant environmental impacts.

**Conclusions:**

Persistent and geographically uneven gender disparities in life expectancy underscore the necessity for localized, gender-sensitive interventions targeting male mortality and environmental health risks.

## Introduction

Life expectancy is a cornerstone indicator of population health, reflecting the cumulative impacts of socio-economic development, healthcare access, environmental quality, and individual behaviors. Globally, women tend to outlive men [1]—a phenomenon attributed to biological advantages (such as hormonal protection) and differences in lifestyle and risk behaviors [2,3].

Recent estimates confirm that the female–male life expectancy gap continues to be substantial, despite its fluctuation over the century [4]. In 2021, the global average life expectancy was 73.8 years for women and 68.4 years for men—a difference of 5.4 years [2]. Evidence also indicates that the gap is strongly influenced by global and regional shocks. For instance, the COVID-19 pandemic disproportionately increased male mortality in many settings, contributing to a temporary widening of the gap [5–7]. In addition, regional crises—such as armed conflict [8], political instability (including female’s political participation [9], economic collapse [10], environmental disasters [11,12], and disease outbreaks [13,14]—have had pronounced localized effects on survival patterns. These events often exacerbate health system weaknesses, restrict access to care, and deepen existing gender-based vulnerabilities, contributing to fluctuations in the life expectancy gap across countries and regions.

While global and national trends provide valuable context, growing evidence indicates that national-level averages can obscure significant subnational disparities in health and mortality outcomes. This limitation is particularly relevant for understanding gender gaps in life expectancy. Thailand, the focus of this study, illustrates this issue clearly. National-level estimates from 2019−2022 show that the female–male life expectancy gap in Thailand ranges from 7.6 to 7.8 years [15]—substantially wider than in many high-income countries [16]. However, in 2021, provincial-level data reveal even greater variation: in some provinces, the gap reaches 8.9–9.5 years, while in others it narrows to just 5.7–6.5 years [15]. These disparities could have been further exacerbated by recent mortality shocks, particularly during the COVID-19 pandemic, which disproportionately increased male mortality [6,7,14].

This study is motivated by two key gaps in existing literature. First, with a few notable exceptions [17,18], relatively little research has examined gender disparities in life expectancy in middle-income or developing countries, where structural inequalities may amplify health risks. Among the limited body of work, only one study has explicitly analyzed the gendered effects of the COVID-19 pandemic on mortality trajectories [5]. Second, even fewer have generated subnational life tables to examine within-country variation in gendered survival—a limitation driven partly by data availability.

To address these gaps, our study constructs life tables for all 77 provinces in Thailand over a nine-year period (2015–2023), offering a detailed account of survival patterns and gender gaps across regions. We focus on three key indicators: life expectancy at birth ($e_{0}$), life expectancy at age 65 ($e_{65}$), and the probability of survival from age 20–65—a proxy for adult mortality risk. Specifically, we address the following questions: (1) What are the levels of $e_{0}$ and $e_{65}$ for females and males across provinces, and how do they compare with national averages? and (2) How have gender differences in these indicators evolved over time, particularly during the COVID-19 period?

Although not the primary focus of this study, we also explore potential drivers of regional variation in the gender gap by examining the correlation of provincial gap estimates with male and female life expectancy and with ambient air pollution levels. This province-level analysis aims to fill an important evidence gap and support more targeted health and development strategies in Thailand and comparable settings.

## Materials and methods

### Data sources

This retrospective study covers all 77 Thai provinces over the period from 2015 to 2023. Annual population and mortality data were obtained from the Bureau of Registration Administration under Thailand’s Ministry of Interior (MOI). Both datasets are derived from Thailand’s civil registration system and are available by single year of age, sex, province, and calendar year.

### Population data

Annual mid-year population counts were used to represent the population at risk. Population data are available by age, sex, year, and province of registration and were used both as exposure denominators in the mortality models and as weights in the aggregation of provincial estimates to the national level.

The population data include Thai and non-Thai individuals recorded in Thailand’s household registration system. The “non-Thai” population refers to individuals who do not hold Thai nationality but are registered in the system, including long-term residents from ethnic minority and migrant groups, foreign spouses of Thai citizens registered in their spouse’s household, and children born in Thailand to non-Thai parents who do not acquire Thai nationality by birth. As non-Thais constitute approximately 1.4% of the total population, their inclusion has negligible effect on the mortality and life table estimates.

### Mortality data

Mortality data, covering Thai and non-Thai deaths, includes individual-level registered deaths occurring between January 1, 2015, and December 31, 2023, with information on age, sex, province of registration, and date of death. No adjustments were made for potential delays in death reporting, as previous evidence indicates that the reporting delays accounted for only 0.03% of deaths over a 12-year period [19].

The completeness of Thailand’s official death registration system has improved significantly over time. According to the National Survey of Population Change in 2015−16, 99.3% of deaths were officially registered [20]. Given this high level of completeness, registered deaths are considered a reliable basis for estimating age- and sex-specific mortality patterns at both national and provincial levels.

### PM2.5 exposure data

PM2.5 exposure data were obtained from the public download portal of the Office of Space Technology Development and Geo-Informatics (GISTDA) PM2.5 monitoring system. At the time of data retrieval, the only publicly available province-level dataset covered calendar year 2024. Twelve monthly files (January–December 2024) were combined to construct a daily province-level time series, from which annual exposure was computed as the mean of daily concentrations by province. PM2.5 concentrations are reported in µg/m³ and are derived from satellite observations integrated with ground monitoring data. Because PM2.5 exposure was available only for 2024, it is treated as a cross-sectional indicator of relative provincial exposure rather than a time-varying covariate.

### Statistical model

A large body of literature models subnational variation in mortality by treating the observed number of deaths as count data and assuming a Poisson distribution, while accounting for systematic effects of age, calendar time, and geography on mortality levels and age profiles [21,22].

We fit generalized additive model (GAMs) to death counts using a Poisson likelihood and a log link. Let $D_{a,t,p}$ denote the observed number of deaths at age a in year t and province p (estimated separately by sex) and let $pop_{a,t,p}$ denote the corresponding exposure (population at risk/person-years). We assume


$$\begin{array}{c} D_{a,t,p}\sim \text{Poisson}\left(\lambda_{a,t,p}\right), \end{array}$$

where $\lambda_{a,t,p}$ is the expected number of deaths. To adjust for differences in exposure across cells, we include $𝐥𝐨𝐠\left(pop_{a,t,p}\right)$ as an offset and model


$$\begin{array}{c} \text{log}\left(\lambda_{a,t,p}\right)=\text{log}\left(pop_{a,t,p}\right)+\eta_{a,t,p} \end{array}$$

This implies $\lambda_{a,t,p}=pop_{a,t,p}\text{exp}\left(\eta_{a,t,p}\right)$, so $\text{exp}\left(\eta_{a,t,p}\right)$ can be interpreted as the modeled mortality rate for the relevant age interval.

The linear predictor is specified to allow flexible age patterns that vary over time and across geography:


$$\begin{array}{c} \eta_{a,t,p}=f_{t}(a)+g_{r(p)}(a)+\gamma_{t,p} \end{array}$$

where $f_{t}(a)$ is a year-specific smooth function of age (a separate smooth age profile for each year), $g_{r(p)}(a)$ is a region-specific smooth function of age (a separate smooth age profile for each region r to which province p belongs), and $\gamma_{t,p}$ is a parametric fixed effect for each year–province combination. Conceptually, $f_{t}(a)$ captures temporal evolution in the age pattern of mortality, while $g_{r(p)}(a)$ captures systematic regional differences in age-specific mortality profiles. The year–province interaction $\gamma_{t,p}$ absorbs additional province-specific annual deviations in overall mortality level beyond what is explained by the smooth age-profile components. This perspective can be viewed as a flexible extension of Lee–Carter-type approaches to modelling age-specific mortality, adapted to subnational settings and richer covariate structures [23].

Smooth functions were represented using cubic regression splines. For each year- and region-specific age smooth, the basis dimension was set to k=15. Smoothing parameters were estimated by Restricted Maximum Likelihood (REML) to balance fit and smoothness. Model diagnostics were used to evaluate basis adequacy and fit, including residual patterns over age and year and checks for overdispersion. Sensitivity analyses with alternative basis dimensions (k=10,20) yielded very similar goodness-of-fit metrics and substantively similar fitted age profiles; therefore, the final specification was guided by visual diagnostics and demographic plausibility.

Models were fitted separately by sex. In both models, we excluded infants (age 0) and restricted the analysis to ages 1–85. From the fitted GAMs, we obtained age-specific mortality rates at the provincial level by sex and year via


$$\begin{array}{c} {\hat{m}}_{a,t,p}=\text{exp}\left({\hat{\eta }}_{a,t,p}\right) \end{array}$$

National age-specific mortality rates were derived as population-weighted aggregates of provincial estimates using age–sex–year-specific exposures as weights.

### Uncertainty estimation

Uncertainty in age-specific mortality rates was derived from the estimated covariance matrix of the GAM. For each province–year–sex–age combination, an approximate 95% uncertainty interval for the fitted mortality rate was computed as:


$$\begin{array}{c} {\hat{m}}_{x}\pm \text{1}.\text{96}\cdot SE({\hat{m}}_{x}) \end{array}$$

where $SE({\hat{m}}_{x})$ denotes the standard error of the fitted rate. Lower and upper mortality schedules constructed from these bounds were then propagated through the full life table calculations to obtain corresponding uncertainty intervals for $e_{0}$, $e_{65}$, and $\backslash {(}_{45}p_{20}$. The same oldest-age adjustment procedure was applied consistently to the lower and upper mortality schedules to ensure coherent uncertainty propagation at advanced ages. For brevity, we report uncertainty estimates only for 2023 in Supplementary Materials. Uncertainty levels in other years were comparable to those observed in 2023.

### Life table computations

The age- and sex-specific mortality rates estimated from the GAM were used to construct period life tables for each province and year, following standard procedures (Preston et al., 2001) [24]. We produced complete life tables with single-year age intervals, starting at age 0 and closing with an open-ended interval at ages 101 + .

Because all age groups are single years, mortality rates were converted to probabilities of death ($q_{x}$) using


$$\begin{array}{c} q_{x}=\frac{m_{x}}{\text{1}+(\text{1}-a_{x})\;m_{x}} \end{array}$$

where $a_{x}$ is the average fraction of the interval lived by those who die within the interval. For all ages except infancy and the open-ended interval, we set $a_{x}=\text{0}.\text{5}$ (deaths occur on average halfway through the year). For age 0, $a_{\text{0}}$ was computed using the Keyfitz empirical approximation ($a_{\text{0}}=\text{0}.\text{0}\text{7}+\text{1}.\text{7}m_{\text{0}}$) [25]. For the open-ended age interval, $a_{x}$ was set to the inverse of the death rate in that interval.

All remaining life table functions, including survivorship ($l_{x}$), person-years lived ($L_{x}$), life expectancy at birth ($e_{0}$) and at age 65 ($e_{65}$), were derived using standard demographic relationships. Survival between ages 20 and 65 was defined as the conditional survival probability—the proportion surviving from exact age 20 to exact age 65:


$$\begin{array}{c} \text{4}\text{5}p_{\text{2}\text{0}}=\frac{l_{\text{6}\text{5}}}{l_{\text{2}\text{0}}} \end{array}$$

All life table computations were implemented consistently across provinces, years, and sexes to ensure comparability of the resulting indicators. National life tables and derived indicators were constructed from aggregated national mortality schedules.

### Treatment of specific age groups

Special consideration was given infant mortality due to its distinct dynamics relative to mortality at older ages. Infant mortality (age 0) was not included in the smooth age functions, as its shape and determinants differ from those at ages 1 and above. Operationally, age 0 was treated as a separate age group, and the observed age-0 mortality rate – computed using registered infant deaths and the mid-year population at age 0 as the exposure denominator – was used directly rather than smoothed. This approach is deemed appropriate because Thailand’s vital registration system is generally considered to be of high quality, and under current Thai mortality levels, variation in $m_{\text{0}}$has a negligible impact on overall life expectancy.

To assess the robustness of life expectancy estimates to alternative treatments of infant mortality, we conducted a sensitivity analysis examining different handling of age-0 mortality and infant $a_{0}$ assumptions. To illustrate the limited influence of infant mortality under current Thai mortality conditions, we note that the minimum age-0 exposure in our province-year series is 554 (men) and 555 (women). Even under a relatively high hypothetical mortality rate for Thailand at age 0 ($m_{0}$=0.01), the standard deviation of the rate implied by binomial variation is small; three standard deviations correspond to a change on the order of 6 × 10 ⁻ ⁵ in the mortality rate, which has a negligible effect on the life table and on $e_{0}$. For life table computations, we follow the Chiang approach, and for age 0 we estimate $a_{0}$ using the standard Keyfitz adjustment $a_{0}=0.07+1.7m_{0}$. Using alternative infant $a_{0}$ approximations (e.g., Preston-type formulations) produces negligible differences in $e_{0}$ under Thai mortality levels. See Scherbov and Ediev (2011) for additional resource of life table accuracy for small populations [26].

Mortality rates at advanced ages also require special treatment. In particular, mortality rates at ages 85 and above often show a paradoxical decline, a phenomenon associated with data limitations such as age misreporting and declining data quality at older ages [27,28]. To address this issue, we applied a logistic adjustment to the oldest-age mortality schedule, generating the probability of death ($q_{x}$) on the logit scale. Specifically, for each province–year–sex combination, we fitted a logistic relationship to $𝐥𝐨𝐠𝐢𝐭(q_{x})$ over ages 65–84, an age range for which mortality patterns are generally well observed and linear in logits. The fitted model was then used to predict $q_{x}$ for ages 85–100, producing a smooth and monotonic extension of the mortality schedule at the oldest ages. The transition from observed to adjusted mortality occurs at age 85 as a hard threshold, but continuity is ensured because the adjustment is anchored to the immediately preceding observed ages. For the terminal open-ended age interval, we set $q_{101+}=1$.

To evaluate the plausibility and impact of the oldest-age extrapolations, we conducted a robustness check comparing life expectancy at age 65 ($e_{65}$) derived with and without the oldest-age extension (see Supplementary S1 Fig). In most cases, life expectancy without adjustment is strongly overestimated. As an additional check, we cross-checked our approach using the constrained extrapolation method [29], in combination with life expectancy estimates derived from previously proposed approaches [28,30] for the oldest old population (results not shown). While these alternative methods produced broadly consistent results, in line with our logistic regression estimates, they also showed inherent instability, with frequent outliers, due to their sensitivity to fluctuations in population growth rates. We therefore retained the logistic adjustment for its robustness and interpretability.

**Fig 1:**
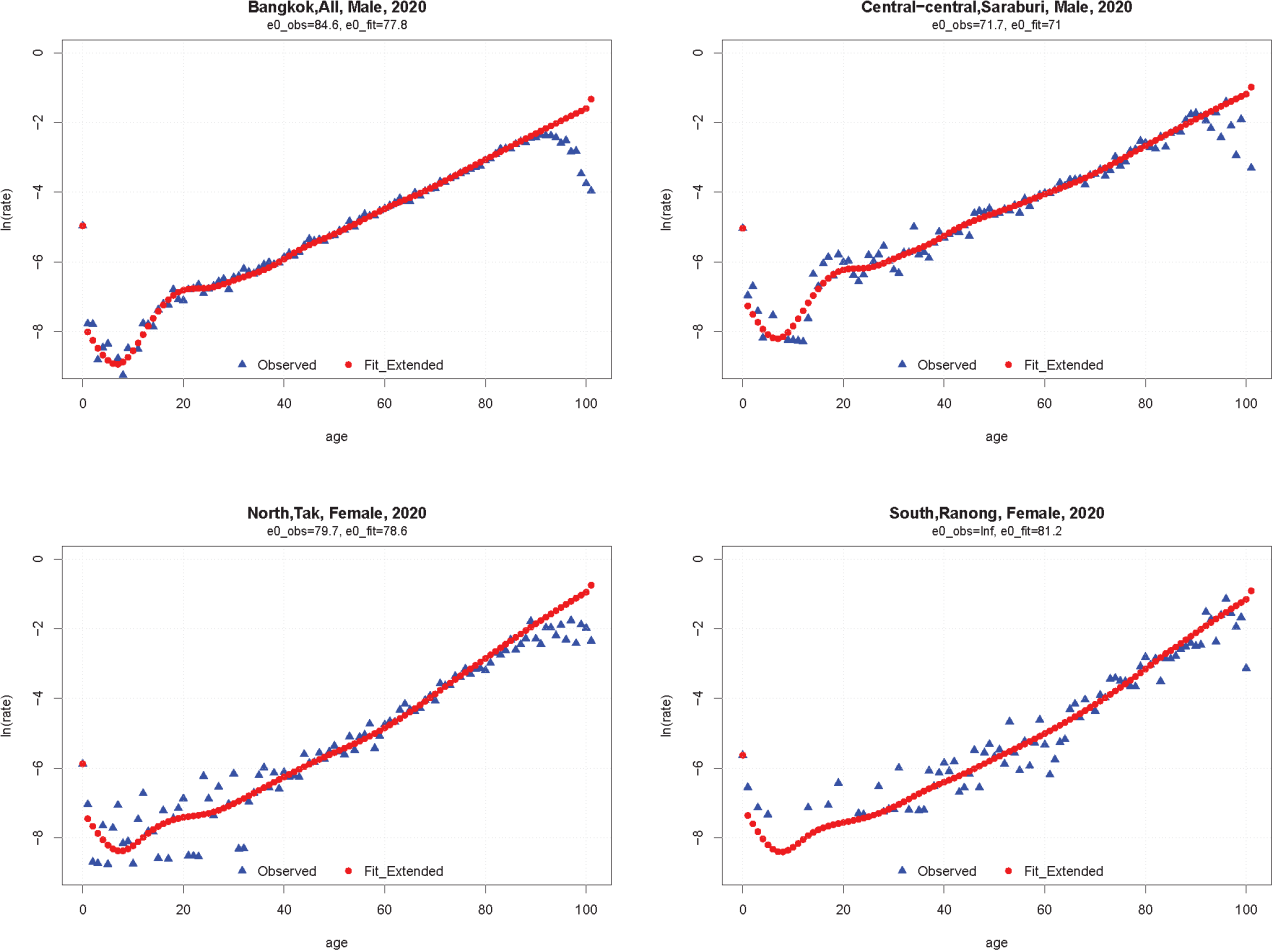
Observed and fitted mortality rates for selected Thai provinces in 2020.

Fig 1 illustrates the extension of mortality rates at older ages and the overall fit of the model to observed mortality rates for ages 1–85, using selected provinces with different population sizes to highlight associated uncertainty. For illustration, we present four cases: Bangkok (males, 2020), Saraburi (males, 2020), Tak (females, 2020), and Ranong (females, 2020). In some provinces—such as Ranong for females—mortality rates in several age groups are recorded as zero because no deaths were registered. In these situations, the regional effect becomes particularly important for producing stable and accurate mortality-rate estimates.

### Ethical considerations

As this study relies on publicly accessible de-identified secondary data collected through official channels, ethical approval was not required. All data handling complied with relevant national data protection guidelines.

## Results

Our analysis of Thai provincial data from 2015 to 2023 reveals significant temporal trends and regional disparities in survival outcomes. In the following subsections, we detail the trends in life expectancy at birth ($e_{\text{0}}$) and at age 65 ($e_{\text{6}\text{5}}$), the evolution of gender gaps over time, discuss the probability to survive from age 20 to age 65, the impact of the COVID‐19 pandemic, and possible drivers behind the gender gap variation. The complete set of results incorporated into an interactive map of Thai provinces could be found in online repository at https://sergio007.shinyapps.io/intmap/.

### National trends in life expectancy at birth (e₀)

In Table 1, between 2015 and 2023, Thailand’s national estimates reveal a steady improvement in life expectancy at birth until 2020. In 2015, life expectancy at birth was 79.6 years for females and 72.6 years for males. By 2020, these figures peaked at around 80.7 years for females and 73.4 years for males, coinciding with the country’s early and effective response to the COVID-19 pandemic such as physical distancing, mask wearing, hand hygiene, widespread testing, and public health communication [31].

**Table 1:** National and province-level distribution of life expectancy at birth ($e_{0}$) by sex, Thailand, 2015–2023.

	2015	2016	2017	2018	2019	2020	2021	2022	2023
**Female**									
Thailand	79.6	79.5	80.1	80.4	80.2	80.7	79.5	79.2	80.1
Provincial minimum $e_{\text{0}}$	77.0	76.7	77.2	77.6	77.6	78.0	75.4	76.0	77.9
Provincial maximum $e_{\text{0}}$	83.6	83.3	83.4	83.5	83.2	85.2	83.2	82.7	83.5
Provincial difference in $e_{\text{0}}$	6.6	6.6	6.2	5.9	5.6	7.1	7.8	6.7	5.6
**Male**									
Thailand	72.6	72.3	72.9	73.2	72.8	73.4	71.8	71.5	72.6
Provincial minimum $e_{\text{0}}$	68.8	68.8	69.8	70.4	69.1	70.2	68.8	68.7	69.3
Provincial maximum $e_{\text{0}}$	76.6	76.4	77.1	77.4	77.2	77.8	75.3	76.1	77.0
Provincial difference in $e_{\text{0}}$	7.8	7.6	7.2	7.0	8.1	7.6	6.5	7.4	7.7
Gender gap in national $e_{\text{0}}$	7.0	7.2	7.2	7.2	7.4	7.3	7.7	7.7	7.5

***Notes:*** *(i) Minimum/maximum refer to the lowest/highest provincial* $e_{\text{0}}$ *within each year. (ii) Provincial difference in* $e_{\text{0}}$ *is the difference between provincial maximum and minimum* $e_{\text{0}}$*. (iii) gender gap in national* $e_{\text{0}}=\overset{F}{\underset{\text{0}}{e}}-\overset{M}{\underset{\text{0}}{e}}$*.*

However, this progress was disrupted during 2021–2022, when Thailand experienced significant waves of COVID-19. The confirmed COVID-related death toll reached 21,693 in 2021 and 11,971 in 2022 [32], contributing to the decline in life expectancy during those years. Female life expectancy dropped to 79.2–79.5 years, while male life expectancy dropped to 71.5–71.8 years-reversing the gains achieved in the preceding years. For 2023, province- and sex-specific $e_{0}$ estimates are reported with 95% uncertainty intervals derived from mortality uncertainty (Supplementary S1 Table).

### Provincial trends in life expectancy at birth

**Fig 2:**
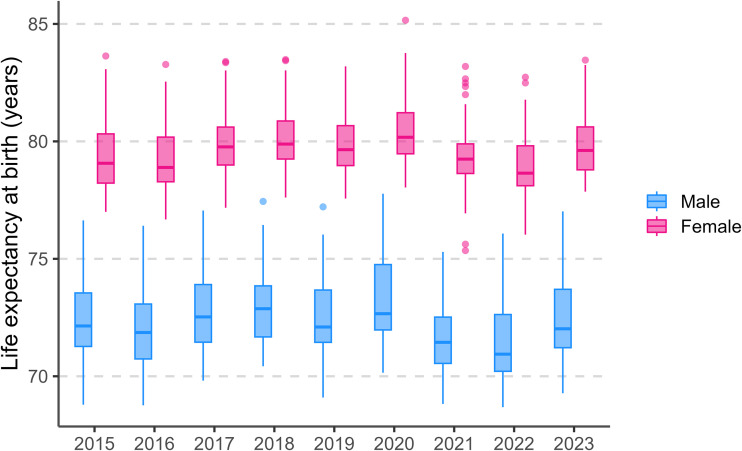
Provincial variation in life expectancy at birth (${𝐞}_{0}$) in Thailand, 2015–2023, by sex. *Boxplots summarize the distribution of provincial estimates each year, the length of box shows the interquartile range (IQR), the horizontal line in the IQR represents the median* ${𝐞}_{0}$ across provinces, the whiskers indicate the minimum value at 1.5*IQR of the 25^*th*^ percentile, and the maximum value at 1.5*IQR of the 75^*th*^ *percentile, and the dots denote outlying provinces with unusually low or high* ${𝐞}_{0}$*.*

Fig 2 presents province-level distributions of life expectancy at birth ($e_{\text{0}}$) by sex from 2015 to 2023 using box-and-whisker plots. Across provinces, the median $e_{\text{0}}$ follows a temporal pattern consistent with national trend. The boxplots also highlight persistent spatial inequality in $e_{\text{0}}$, indicated by the width of the interquartile ranges (IQRs) and the length of the whiskers. In most years, the male distributions are more dispersed than the female distributions, suggesting greater interprovincial heterogeneity in mortality conditions among men.

Based on Table 1 and its details provided in online repository, the difference between the highest and lowest provincial $e_{0}$ ranged from 5.6–7.8 years among women and 6.5–8.1 years among men. Spatial clustering is evident. Across the study period, Bangkok and surrounding provinces were in general at or above the national average $e_{0}$. A high share of provinces in Southern Thailand also remained at or above the national average (typically 11–12 of 14 provinces).

In 2020—the year with the highest national $e_{0}$ for both gender—the highest $e_{0}$ for women was observed in Phatthalung (85.2 years) and the lowest provincial $e_{0}$ occurred in Nakhon Phanom (78.0 years) for women. For men, the highest provincial $e_{0}$ was observed in Bangkok (77.8 years), and the lowest provincial $e_{0}$ occurred in Kalasin (70.2 years). Bangkok with surrounding provinces and 11 out of 14 provinces in the South exhibited female life expectancy levels at or above the national average. Likewise, Bangkok with surrounding provinces and 12 out of 14 provinces in the South (excluding Narathiwat and Yala) were at or above the male life expectancy at the national average.

In contrast, a clear clustering of lower longevity is observed in the Northeast and North. In 2020, 19 out of 20 provinces in the Northeast (for both sexes), and 14 of 17 provinces in North for men, and 15 of 17 provinces in the North for women had life expectancy levels below national average (See maps in online repository).

We also observed that several provinces with above-average life expectancy such as Bangkok and surrounding provinces, Chon Buri, and Ranong, exhibited signs of recovery in 2022–2023, suggesting a possible rebound in survival outcomes following the pandemic’s peak (See maps in online repository).

These spatial patterns provide a foundation for further examination of gender gaps and regional disparities in life expectancy, particularly in the context of pandemic-related shocks.

### Gender gap in life expectancy at birth

Analysis of national data from 2015 to 2023 reveals a persistent gender gap in life expectancy at birth, with females consistently outliving males (Table 1). In 2015, the national-level gender gap in life expectancy at birth was 7.0 years. This gap widened slightly to 7.3 years by 2020. The gap increased more markedly in 2021—when COVID-19 had its most severe impact on Thailand—reaching 7.7 years. By 2023, the gender gap had narrowed slightly to 7.5 years, though it remained higher than the pre-pandemic level. This trend mirrors patterns observed in the United States, where COVID-19 was identified as a major contributor to the widening gender gap in life expectancy [7].

All province-year life expectancy estimates and derived gender-gap measures are available in the online repository. Using these province-year estimates, we find substantial heterogeneity at the provincial level, although the overall trajectory aligns with the national pattern. In 2015 the widest gender gap was recorded in Singburi (8.9 years), while the narrowest was in Loei (5.3 years). By 2020, Phatthalung emerged as the province with the widest gender gap (9.3 years), while Satun exhibited the narrowest (4.3 years). In 2022, Phatthalung again reported the highest gender gap at 9.5 years, suggesting that the pandemic may have disproportionately impacted male survival in the province. In contrast, Narathiwat had the lowest gender gap at 5.9 years.

**Fig 3:**
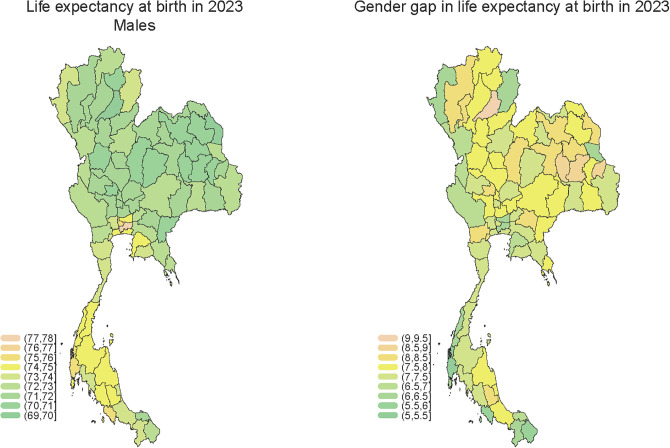
Provincial patterns of male life expectancy at birth and gender gap in life expectancy at birth at birth, Thailand, 2023. Maps were produced using administrative boundary data provided by geoBoundaries and made available under the Creative Commons Attribution 4.0 (CC BY 4.0) license [33].

Fig 3 visualizes these disparities in 2023, with the left panel showing male life expectancy at birth and the right panel illustrating the gender gap. Provinces in the North and Northeast generally exhibit lower male life expectancy and larger gender gaps, while Southern provinces such as Yala, Pattani, and Narathiwat report both higher male life expectancy and narrower gender gaps. These spatial patterns reinforce the need to explore how socio-economic, cultural, behavioral, and environmental factors intersect to shape gendered survival outcomes—particularly during and after pandemic-related health system disruptions.

**Fig 4:**
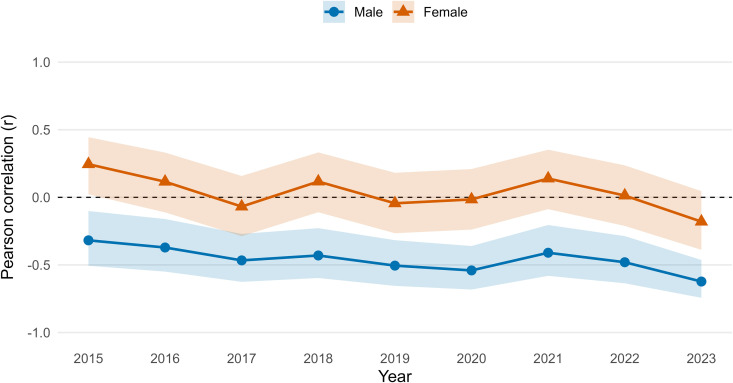
Year-specific association between provincial life expectancy at birth and the gender gap in life expectancy in Thailand, 2015–2023. *For each year, we calculated Pearson’s correlation coefficient (*𝐫*) across provinces between male (blue) or female (orange) provincial life expectancy at birth (*${𝐞}_{0}$*) and the provincial gender gap in life expectancy at birth (female – male,* $\overset{F}{\underset{0}{𝐞}}-\overset{M}{\underset{0}{e}}$*). Shaded bands show 95% confidence intervals for* 𝐫 *based on Fisher’s z transformation.*

An analysis of the correlation patterns provides further insight into the dynamics underlying these gender differences (Fig 4). From 2015 to 2023, the correlation between male life expectancy at birth and the gender gap was consistently strong and negative, reaching −0.7 in 2023. This indicates that lower male life expectancy is strongly associated with a wider gender gap, suggesting that male-specific mortality factors are a primary driver of the disparity. In contrast, the correlation between female life expectancy at birth and the gender gap was statistically insignificant and fluctuated around zero, implying that variations in female longevity play a minimal role in shaping the gap.

### Trends in life expectancy at age 65 and gender gap

As shown in Table 2 with full province-year results in the online repository, the geographic distribution of life expectancy at age 65 is broadly consistent with the spatial clustering observed for life expectancy at birth (Table 1). National life expectancy at age 65 ($e_{\text{6}\text{5}}$) improved gradually before the COVID-19 pandemic, with noticeable differences between females and males. In 2015, the national $e_{\text{6}\text{5}}$ was 19.6 years for females and 17.2 years for males—a gender gap of around 2.4 years. The life expectancy at age 65 increased steadily and peaked in 2020, when women enjoyed a life expectancy of 20.3 years and men 17.8 years, resulting in an increased gap of 2.6 years at age 65. However, the relaxation of state responses to COVID-19 during 2021–2022 led to a shift in the trend, reversing earlier improvements. In 2022, life expectancy at age 65 declined to 19.1 years for females and 16.3 years for males, resulting in a gender gap of 2.9 years. This widening occurred not because women were unaffected, but because male life expectancy declined more sharply, reflecting differential vulnerability to COVID-19 related mortality [5,6].

**Table 2:** National and province-level distribution of life expectancy at birth ($e_{65}$) by sex, Thailand, 2015–2023.

	2015	2016	2017	2018	2019	2020	2021	2022	2023
**Female**									
Thailand	19.6	19.4	20.0	20.2	20.0	20.3	19.6	19.1	19.9
Provincial minimum $e_{\text{6}\text{5}}$	17.7	17.3	17.7	17.9	18.1	18.2	17.3	17.3	18.1
Provincial maximum $e_{\text{6}\text{5}}$	22.7	22.4	22.5	22.7	22.4	23.9	22.7	21.7	22.4
Provincial difference in $e_{\text{6}\text{5}}$	5.0	5.1	4.8	4.7	4.3	5.8	5.4	4.4	4.2
**Male**									
Thailand	17.2	17.0	17.5	17.8	17.4	17.8	16.7	16.3	17.0
Provincial minimum $e_{\text{6}\text{5}}$	15.3	15.1	15.8	16.2	15.5	16.0	14.7	14.7	15.4
Provincial maximum $e_{\text{6}\text{5}}$	19.3	19.1	19.7	20.1	19.8	20.2	18.4	18.6	19.3
Provincial difference in $e_{\text{6}\text{5}}$	4.1	4.0	3.9	3.9	4.4	4.1	3.7	3.9	3.9
Gender gap in national $e_{\text{6}\text{5}}$	2.4	2.4	2.5	2.4	2.6	2.5	2.9	2.8	2.9

***Notes:*** *(i) Minimum/maximum refer to the lowest/highest provincial* $e_{\text{6}\text{5}}$ *within each year. (ii) Provincial difference in* $e_{\text{6}\text{5}}$ *is the difference between provincial maximum and minimum* $e_{\text{6}\text{5}}$*. (iii) gender gap in national* $e_{\text{6}\text{5}}=\overset{F}{\underset{\text{6}\text{5}}{e}}-\overset{M}{\underset{\text{6}\text{5}}{e}}$*.*

Importantly, these trends were not uniform across regions. In 2020, at the provincial level, Phatthalung recorded the highest female life expectancy at age 65 (23.9 years), while Nakhon Phanom reported the lowest (18.2 years). For males, Bangkok (20.2 years) and southern provinces such as Satun (19.3 years) and Phuket (19.1 years) exhibited relatively high life expectancy at age 65. In contrast, Kalasin in the Northeast and Lamphun in the North reported the lowest male values (both 16.0 years).

Provincial heterogeneity is also evident in the gender gap at age 65. In 2020, the gap was most pronounced in Phatthalung at 4.9 years, while Tak and Mukdahan had the narrowest gaps (0.8 years and 1.0 year, respectively). These regional disparities in both absolute survival and gender gaps suggest underlying structural differences in healthcare access [34], prevalence of chronic conditions [35], and behavioral health risks [36], all of which likely shaped the pandemic’s impact on elderly survival.

### Probability to survive from age 20 to age 65

Although life expectancy at birth and the probability of surviving from age 20 to age 65 are closely related, the latter provides insights into adult mortality and workforce health (data is available in the online repository or Supplementary S3 Table for 2023).

At the national level, the probability of surviving from age 20 to age 65 in Thailand exhibited a generally stable pattern over time, with gradual improvements before the COVID-19 pandemic and a notable decline in 2021. In 2020, when survival probabilities peaked, the national average stood at 88.3% for females and 74.6% for males. However, the impact of the pandemic in 2021–2022 led to a decline, with survival probabilities dropping to 86.9% for females and 72.0% for males, before partially recovering in 2023. While these shifts are modest, they align with broader evidence that male survival was more adversely affected by the pandemic, reinforcing observed gender disparities in life expectancy [5,6].

At the provincial level, our data reveal notable variability in the probability of surviving from age 20 to age 65 years across Thailand. While the overall time trend aligns with national averages, certain provinces consistently outperform or underperform compared to the national survival rate.

Among the provinces with the high survival probabilities, Bangkok consistently recorded values well above the national average. In 2020—when survival rates were at their peak nationally—Bangkok reported probabilities of approximately 91.2% for females and 80.9% for males, higher than the national figures. In 2021, Bangkok experienced a decline, with survival dropping to 88.4% for females and 74.9% for males, though it remained above the national trend.

Conversely, Sa Kaew—a relatively poor eastern province bordering Cambodia—and Phrae—a small mountainous province in northern Thailand—consistently exhibited some of the lowest survival probabilities from age 20–65. In 2020, Sa Kaew reported 85.6% survival for females, while Phrae recorded 69.5% for males. By 2022, these figures declined to 83.5% for females in Sa Kaew and 68.1% for males in Phrae. These provinces remained in the lower quintile of survival across all years in the study period, reflecting persistently lower midlife survival outcomes.

### Association between PM2.5 and life expectancy

Analysis of provincial PM2.5 levels and life expectancy across Thailand revealed notable regional disparities. Urban centers such as Bangkok recorded average annual PM2.5 concentrations exceeding 20 μg/m³, while many southern provinces maintained lower levels, often below 15 μg/m³. Northern provinces exhibited elevated PM2.5 levels, largely attributable to seasonal agricultural burning and industrial activity.

**Fig 5:**
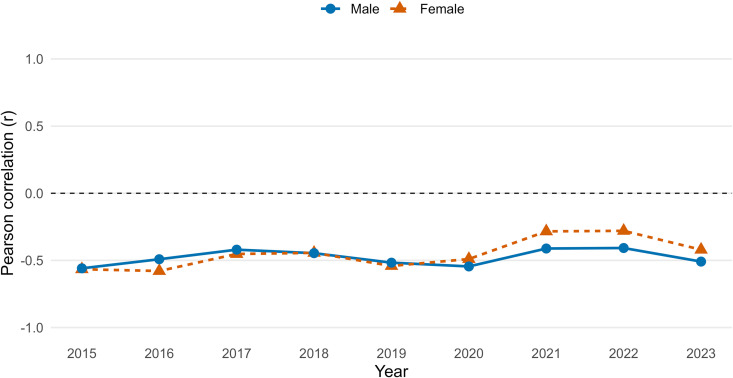
Year-specific correlation between regional PM2.5 exposure and life expectancy at birth (${𝐞}_{0}$) in Thailand, 2015–2023. *Lines show Pearson’s correlation coefficient (*𝐫*) across regions between regional PM2.5 and regional life expectancy at birth (*${𝐞}_{0}$*) for males (blue) and females (orange), computed using complete cases only. The horizontal dashed line indicates* 𝐫=0*.*

To examine this relationship more systematically, we computed Pearson correlation coefficients between recorded PM2.5 values (from 2024—the only available provincial dataset) and average female life expectancy at birth from 2015 to 2023. The results, presented in Fig 5, reveal a moderate to strong negative correlation, with coefficients ranging from –0.5 to –0.6. This indicates that provinces with higher levels of PM2.5 tended to have lower female life expectancy. During the height of the COVID-19 pandemic (2021–2022), the correlation between the decline in life expectancy and PM2.5 levels was the weakest over the study period.

## Discussion

This study provides new empirical evidence on gender disparities in life expectancy across Thailand’s 77 provinces from 2015 to 2023, with particular attention to provincial-level deviations from national patterns. By constructing subnational life tables using national vital registration data, we systematically examine temporal shifts in gender gaps during the COVID-19 pandemic and explore the role of socio-economic, healthcare, and environmental factors, especially PM2.5 exposure, in shaping survival inequalities. These findings address the underexplored issue of within-country gender gaps in middle-income settings.

Between 2015–2019, Thailand experienced modest but steady improvements in life expectancy. Life expectancy at birth increased from 79.6 to 80.2 years for females and from 72.6 to 72.8 years for males. Similarly, life expectancy at age 65 years rose from 19.6 to 20.3 for females, and from 17.2 years to 17.8 years for males. These gains led to a slight widening of the gender gap from 7.0 to 7.4 years in life expectancy at birth, and from 2.4 to 2.5 years at age 65. The findings are consistent with broader trends observed across Southeast Asia [37], where men consistently live shorter than women [38,39].

However, this trajectory was disrupted by the COVID-19 pandemic. Our findings show that the widening gender gap was largely driven by excess male mortality, with the COVID-19 pandemic acting as an amplifying factor between 2020–2023. In 2020, Thailand initially gained survival advantages through effective public health responses such as physical distancing, mask wearing, hand hygiene, and public health communication [31] which contributed to a modest increase of 0.5–0.6 years in life expectancy at birth and temporarily stabilized the gender gap. However, by 2022, the relaxation of these measures coincided with a sharper decline in male life expectancy (−1.8 years) compared to females (−1.5 years), resulting in the gender gap widening to 7.7 years [2,40]. Similar patterns were observed in life expectancy at age 65, with males experiencing a larger drop. By 2023, male life expectancy rebounded slightly faster than that of females. This pattern is confirmed by our correlation analysis (see Fig 3). These results are consistent with global studies suggesting that men were more vulnerable to COVID-19-related mortality [5,6,41,42].

Our findings of regional life expectancy align with previous studies that documented geographical variation in mortality across Thailand using national vital registration data [35,43]. Provinces in the North and Northeast consistently reported lower life expectancy, a pattern likely driven by structural socio-economic disadvantages. These regions have a higher proportion of older adults and elevated poverty rates [44], both of which are associated with increased risks and reduced access to health-promoting resources [36,43,45].

Unlike provinces in the North and Northeast, where economic disadvantage is associated with lower life expectancy, the Southern region—despite having the highest poverty rate since 2017 [44]—has consistently reported higher life expectancy. This apparent paradox may be attributed to distinct socio-cultural and behavioral factors. Prior research indicates that the southern region—particularly provinces with a higher proportion of Muslim residents—tends to exhibit healthier lifestyles. These include lower levels of alcohol consumption [46], greater intake of fruit and vegetable intake [36], and lower rates of road traffic accidents, particularly in provinces like Satun, Pattani, and Yala [36]. Collectively, these behaviors may contribute to more favorable health outcomes.

Moreover, provinces with predominantly Muslim populations may also experience narrower gender gaps in life expectancy. Among Thai men, approximately 30.4% of total disease burden is attributable to smoking and alcohol consumption, whereas these factors contribute minimally to disease burden among women [47]. In predominantly Muslim provinces—where religious practices discourage such behaviors—male mortality risks may be comparatively lower, contributing to smaller gender gaps in life expectancy observed in provinces such as Narathiwat, Pattani, and Yala. This supports the hypothesis that gender differences in behavioral risk exposures play a significant role in shaping survival disparities.

This spatial heterogeneity in gender gaps may reflect the interaction between biological and behavioral factors. Estrogen provides enhanced immune system, while the presence of two X chromosomes may offer greater genetic resilience [39,42]. In terms of health behavior, Thai women were significantly less likely to smoke across all age groups and regions [36,48]. Between 1990 and 2019, the smoking prevalence among Thai women declined by 46.5%, compared to a 25.5% reduction among men [49]. Furthermore, the proportion of adults engaging in physical activity—especially among those aged 60 and above—increased from 58.6% in 2012 to 73.4% in 2019 [36]. These biological and behavioral advantages underpin the consistent female advantage in life expectancy across all ages. This partly explains the observed resilience of female life expectancy during the pandemic, even as male life expectancy declined more sharply. Nevertheless, the gender gap becomes slightly narrower at older ages, possibly due to selective survival and reduced male–female differences in risk exposure later in life [50].

Moreover, socio-economic status remains a critical determinant of health inequalities. Our findings that provinces with lower socio-economic development, such as many in the Northeast and North of Thailand, tended to exhibit lower life expectancy and wider gender gaps, align with the evidence that low-income and rural areas typically face compounded disadvantages: limited access to quality healthcare, fewer educational opportunities, environmental hazards, and unstable employment [51–53]. Moreover, COVID-19 exacerbated existing socio-economic vulnerabilities. Similar to patterns observed internationally [54,55], regions with higher poverty, weaker health infrastructure, and greater proportions of disadvantaged groups faced disproportionately higher mortality impacts, particularly among men who tend to engage in riskier behaviors, have greater biological vulnerabilities, and are overrepresented in high-risk occupations [5–7,17,41,56].

Variations in healthcare access and quality further contributed to differential survival outcomes across provinces [34,57]. During the COVID-19 pandemic, widespread strain on healthcare systems led to disruptions in essential services for non-communicable diseases (NCDs) such as diagnosis, treatment, rehabilitation, and palliative care [58,59]. Infection control measures, including lockdowns and work-from-home policies [31], may have further limited healthcare access [58], disproportionately affecting male survival risks. Our findings suggest that provinces with stronger healthcare infrastructure, such as Bangkok and surrounding metropolitan areas, exhibited faster recovery in life expectancy trends compared to rural and underserved areas.

Environmental factors, particularly exposure to fine particulate matter (PM2.5), may further influence survival probabilities. Provinces in the North, such as Chiang Mai and Lampang, reported higher levels of PM2.5 concentrations, correlating with elevated mortality from chronic obstructive pulmonary disease (COPD) [60], and lower life expectancy observed in our findings. Long-term exposure to PM2.5 is strongly associated with increased risks of cardiovascular and respiratory mortality [61–64]. Moreover, emerging evidence suggests that although ischemic heart disease and stroke are more prevalent among men [36,65], women may exhibit greater biological vulnerability to the cardiovascular effects of particulate pollution [64], potentially contributing to regional gender differences in survival outcomes.

### Strengths and limitations

A key strength of our study is the use of detailed provincial data spanning a nine-year period, which allows for an in-depth analysis of temporal trends and regional heterogeneity. However, several limitations must be acknowledged. First, our analysis relies on administrative mortality records, which may be subject to reporting biases that vary by province. Second, although our GAM framework effectively captures nonlinear and spatial effects, residual confounding from unmeasured variables—such as detailed socio-economic indicators—may persist. Third, while we reference existing literature on PM2.5, the absence of direct integration of detailed provincial pollution data limits our ability to quantify its specific contribution to regional survival differences. Furthermore, this study did not aim to determine whether environmental and behavioral effects might be confounded. This is an important direction for future research. Finally, recent studies have raised methodological concerns regarding the use of traditional period life tables for estimating changes in life expectancy during periods of crisis such as pandemics [66]. In such contexts, hybrid life expectancy—combining period and cohort perspectives—may provide a more accurate measure of population survival under rapidly changing mortality conditions.

## Conclusion

This study shows that gender disparities in life expectancy across Thailand’s provinces remain persistent and geographically uneven. The COVID-19 pandemic magnified these differences, particularly where structural and environmental vulnerabilities were greatest. Addressing gendered survival gaps requires attention to local health system capacity, behavioral risk factors, and environmental exposures. Tailored health policies—especially those targeting male health risks and pollution-related burdens—can help reduce inequalities and improve population health equity.

## Supporting information

S1 FigSensitivity of GAM-derived to oldest-old (85+) closure.Boxplots summarize, by year (2015–2023), the difference in regional $e_{\text{6}\text{5}}$ obtained from the GAM-fitted mortality schedule after applying an 85 + logit-closure adjustment compared with the unadjusted GAM-based estimate (Δ$e_{\text{6}\text{5}}$ = adjusted − unadjusted). Male and female results are shown in separate panels; the zero line denotes no effect. Outliers with |Δ$e_{\text{6}\text{5}}$| > 20 years were excluded. The overwhelmingly negative differences highlight the biases in the original data caused by age exaggeration, and emphasize the importance of adjustment to obtain more reliable life expectancy estimates.(PNG)

S1 TableLife expectancy at birth ($e_{0}$) by province and sex, Thailand, 2023.Values shown as mean (lower, upper); bounds are 95% interval limits derived from mortality uncertainty.(DOCX)

S2 TableLife expectancy at age 65 ($e_{65}$) by province and sex, Thailand, 2023.Values shown as mean (lower, upper); bounds are 95% interval limits derived from mortality uncertainty.(DOCX)

S3 TableProbability of surviving from age 20–65 ($20p_{65}$) by province and sex, Thailand, 2023.Values shown as mean (lower, upper); bounds are 95% interval limits derived from mortality uncertainty.(DOCX)
