# Supplementary figures and images for: Survival and life expectancy inequality by gender in Thai provinces: Trends from 2015 to 2023
Source: PLoS One. 2026 May 13;21(5):e0348587. doi: 10.1371/journal.pone.0348587 (PMC13170844; doi:10.1371/journal.pone.0348587)

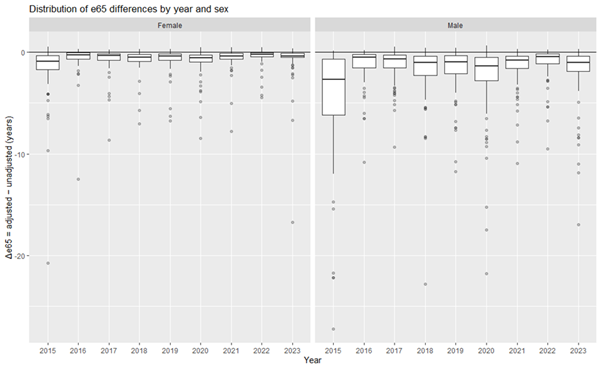

Supplement: S1 Fig — Boxplots summarize, by year (2015–2023), the difference in regional e65 obtained from the GAM-fitted mortality schedule after applying an 85 + logit-closure adjustment compared with the unadjusted GAM-based estimate (Δe65 = adjusted − unadjusted). Male and female results are shown in separate panels; the zero line denotes no effect. Outliers with |Δe65| > 20 years were excluded. The overwhelmingly negative differences highlight the biases in the original data caused by age exaggeration, and emphasize the importance of adjustment to obtain more reliable life expectancy estimates. (PNG) [file pone.0348587.s001.png]
