# Supplementary material for: Survival and life expectancy inequality by gender in Thai provinces: Trends from 2015 to 2023
Source: PLoS One. 2026 May 13;21(5):e0348587. doi: 10.1371/journal.pone.0348587 (PMC13170844; doi:10.1371/journal.pone.0348587)
Supplement: S1 Table — Values shown as mean (lower, upper); bounds are 95% interval limits derived from mortality uncertainty. (DOCX) [file pone.0348587.s002.docx]

**S1 Table.** **Life expectancy at birth (**$\boldsymbol{e}_{\boldsymbol{0}}$**) by province and sex, Thailand, 2023. Values shown as mean (lower, upper); bounds are 95% interval limits derived from mortality uncertainty.**

| **Region** | **Province** | **Female** | **Male** |
| --- | --- | --- | --- |
| **Thailand** | | **80.1 (79.6, 80.7)** | **72.6 (72.0, 73.2)** |
| Bangkok | Bangkok | 83.5 (83.0, 83.9) | 77.0 (76.6, 77.5) |
| Peripheral area | Nakhon Pathom | 81.1 (80.5, 81.8) | 73.7 (73.1, 74.3) |
| Peripheral area | Nonthaburi | 82.6 (82.0, 83.2) | 75.2 (74.6, 75.8) |
| Peripheral area | Pathum Thani | 81.0 (80.5, 81.7) | 74.8 (74.2, 75.4) |
| Peripheral area | Samut Prakan | 81.8 (81.2, 82.4) | 74.7 (74.1, 75.3) |
| Peripheral area | Samut Sakhon | 81.9 (81.2, 82.7) | 74.6 (73.8, 75.3) |
| Central | Ang Thong | 78.6 (77.8, 79.5) | 71.4 (70.5, 72.3) |
| Central | Chai Nat | 79.0 (78.2, 79.8) | 70.7 (69.9, 71.5) |
| Central | Lop Buri | 78.6 (78.0, 79.3) | 71.2 (70.6, 71.9) |
| Central | Phra Nakhon Sri Ayuthaya | 80.0 (79.3, 80.7) | 72.0 (71.4, 72.6) |
| Central | Saraburi | 78.9 (78.2, 79.6) | 71.3 (70.7, 72.0) |
| Central | Singburi | 79.7 (78.8, 80.8) | 72.0 (71.0, 73.0) |
| East | Chachoengsao | 80.1 (79.4, 80.8) | 72.9 (72.3, 73.6) |
| East | Chanthaburi | 80.2 (79.5, 80.9) | 72.9 (72.3, 73.7) |
| East | Chon Buri | 80.8 (80.2, 81.4) | 74.2 (73.7, 74.8) |
| East | Nakhon Nayok | 79.6 (78.7, 80.5) | 72.3 (71.4, 73.3) |
| East | Prachin Buri | 79.9 (79.1, 80.7) | 71.7 (71.0, 72.4) |
| East | Rayong | 80.5 (79.8, 81.2) | 73.1 (72.4, 73.8) |
| East | Sa Kaew | 77.9 (77.2, 78.6) | 70.1 (69.4, 70.8) |
| East | Trat | 80.4 (79.4, 81.5) | 72.7 (71.8, 73.7) |
| North | Chiang Mai | 79.9 (79.5, 80.4) | 71.6 (71.2, 72.1) |
| North | Chiang Rai | 79.7 (79.3, 80.2) | 72.1 (71.6, 72.6) |
| North | Kam Phaeng Phet | 78.4 (77.9, 79.0) | 70.8 (70.2, 71.4) |
| North | Lampang | 79.2 (78.7, 79.7) | 71.6 (71.0, 72.1) |
| North | Lamphun | 79.4 (78.7, 80.1) | 71.1 (70.4, 71.8) |
| North | Mae Hong Son | 79.9 (79.0, 80.9) | 73.7 (72.8, 74.7) |
| North | Nakhon Sawan | 78.6 (78.2, 79.1) | 71.1 (70.6, 71.6) |
| North | Nan | 80.4 (79.7, 81.1) | 73.9 (73.2, 74.6) |
| North | Phayao | 78.3 (77.7, 78.9) | 70.6 (70.0, 71.3) |
| North | Phetchabun | 78.6 (78.1, 79.1) | 70.6 (70.1, 71.1) |
| North | Phichit | 78.9 (78.3, 79.5) | 71.4 (70.7, 72.0) |
| North | Phitsanulok | 79.3 (78.8, 79.8) | 72.2 (71.7, 72.8) |
| North | Phrae | 78.6 (78.0, 79.3) | 69.3 (68.6, 69.9) |
| North | Sukhothai | 78.8 (78.2, 79.3) | 71.2 (70.6, 71.8) |
| North | Tak | 78.7 (78.0, 79.3) | 72.1 (71.5, 72.8) |
| North | Uthai Thani | 79.1 (78.4, 79.9) | 71.7 (71.0, 72.5) |
| North | Uttaradit | 79.0 (78.4, 79.6) | 71.2 (70.5, 71.9) |
| Northeast | Amnat Chareon | 80.3 (79.5, 81.0) | 71.3 (70.5, 72.1) |
| Northeast | Bueng Kan | 79.0 (78.3, 79.8) | 71.0 (70.3, 71.8) |
| Northeast | Buri Ram | 79.6 (79.2, 80.0) | 71.7 (71.3, 72.2) |
| Northeast | Chaiyaphum | 78.3 (77.8, 78.7) | 70.8 (70.3, 71.3) |
| Northeast | Kalasin | 78.8 (78.3, 79.3) | 70.4 (69.8, 70.9) |
| Northeast | Khon Kaen | 79.5 (79.1, 79.9) | 71.2 (70.8, 71.7) |
| Northeast | Loei | 79.6 (79.1, 80.2) | 71.9 (71.4, 72.6) |
| Northeast | Mukdahan | 79.1 (78.4, 79.9) | 72.8 (72.0, 73.6) |
| Northeast | Naha Sarakham | 78.6 (78.2, 79.1) | 70.1 (69.5, 70.6) |
| Northeast | Nakhon Phanom | 78.0 (77.5, 78.6) | 69.9 (69.4, 70.5) |
| Northeast | Nakhon Ratchasima | 80.2 (79.8, 80.5) | 72.3 (71.9, 72.7) |
| Northeast | Nong Khai | 79.1 (78.4, 79.7) | 71.9 (71.3, 72.6) |
| Northeast | Nongbua Lamphu | 78.8 (78.2, 79.5) | 71.5 (70.9, 72.2) |
| Northeast | Roi Et | 79.2 (78.8, 79.7) | 70.5 (70.0, 71.0) |
| Northeast | Sakon Nakhon | 78.5 (78.0, 79.0) | 70.9 (70.4, 71.4) |
| Northeast | Si Sa Ket | 79.5 (79.1, 80.0) | 71.9 (71.4, 72.4) |
| Northeast | Surin | 79.8 (79.4, 80.3) | 72.4 (71.9, 72.9) |
| Northeast | Ubon Ratchathani | 79.4 (79.0, 79.8) | 72.3 (71.8, 72.7) |
| Northeast | Udon Thani | 78.7 (78.3, 79.1) | 70.5 (70.0, 70.9) |
| Northeast | Yasothon | 78.4 (77.8, 79.0) | 70.4 (69.8, 71.1) |
| South | Chumphon | 81.5 (80.8, 82.3) | 74.1 (73.4, 74.8) |
| South | Krabi | 81.3 (80.5, 82.2) | 73.9 (73.1, 74.7) |
| South | Nakhon Si Thammarat | 81.9 (81.4, 82.4) | 74.2 (73.7, 74.8) |
| South | Narathiwat | 77.9 (77.2, 78.5) | 72.3 (71.6, 73.0) |
| South | Pattani | 78.7 (78.0, 79.4) | 72.0 (71.3, 72.7) |
| South | Phangnga | 80.4 (79.5, 81.4) | 75.2 (74.2, 76.2) |
| South | Phatthalung | 83.3 (82.5, 84.1) | 75.0 (74.3, 75.7) |
| South | Phuket | 80.6 (79.8, 81.6) | 74.5 (73.6, 75.4) |
| South | Ranong | 80.7 (79.6, 81.9) | 74.6 (73.5, 75.8) |
| South | Satun | 80.8 (79.9, 81.8) | 75.1 (74.2, 76.1) |
| South | Songkhla | 81.7 (81.1, 82.2) | 73.9 (73.3, 74.4) |
| South | Surat Thani | 82.0 (81.4, 82.6) | 74.6 (74.0, 75.2) |
| South | Trang | 81.8 (81.1, 82.6) | 74.5 (73.8, 75.2) |
| South | Yala | 80.1 (79.3, 80.9) | 73.6 (72.8, 74.4) |
| West | Kanchanaburi | 79.3 (78.7, 80.0) | 72.5 (71.9, 73.1) |
| West | Phachuap Khiri Khan | 81.2 (80.4, 82.0) | 73.9 (73.2, 74.7) |
| West | Phetchaburi | 80.8 (80.1, 81.6) | 73.6 (72.9, 74.4) |
| West | Ratchaburi | 80.3 (79.6, 81.0) | 72.0 (71.5, 72.6) |
| West | Samut Songkhram | 81.3 (80.3, 82.4) | 73.2 (72.2, 74.2) |
| West | Suphan Buri | 79.0 (78.4, 79.7) | 71.4 (70.9, 72.0) |
