# Supplementary material for: Survival and life expectancy inequality by gender in Thai provinces: Trends from 2015 to 2023
Source: PLoS One. 2026 May 13;21(5):e0348587. doi: 10.1371/journal.pone.0348587 (PMC13170844; doi:10.1371/journal.pone.0348587)
Supplement: S2 Table — Values shown as mean (lower, upper); bounds are 95% interval limits derived from mortality uncertainty. (DOCX) [file pone.0348587.s003.docx]

**S2 Table. Life expectancy at age 65 (**$\boldsymbol{e}_{\boldsymbol{65}}$**) by province and sex, Thailand, 2023. Values shown as mean (lower, upper); bounds are 95% interval limits derived from mortality uncertainty.**

| **Region** | **Province** | **Female** | **Male** |
| --- | --- | --- | --- |
| **Thailand** | | **19.9 (19.5, 20.3)** | **17.0 (16.6, 17.3)** |
| Bangkok | Bangkok | 22.2 (21.9, 22.6) | 19.3 (19.0, 19.7) |
| Peripheral area | Nakhon Pathom | 20.4 (20.0, 20.9) | 17.1 (16.8, 17.5) |
| Peripheral area | Nonthaburi | 21.6 (21.2, 22.0) | 18.1 (17.7, 18.5) |
| Peripheral area | Pathum Thani | 20.4 (20.0, 20.8) | 17.8 (17.4, 18.2) |
| Peripheral area | Samut Prakan | 20.9 (20.5, 21.4) | 17.6 (17.3, 18.0) |
| Peripheral area | Samut Sakhon | 21.0 (20.5, 21.6) | 17.7 (17.2, 18.2) |
| Central | Ang Thong | 19.5 (18.9, 20.1) | 16.5 (16.0, 17.0) |
| Central | Chai Nat | 19.3 (18.8, 19.9) | 16.1 (15.7, 16.6) |
| Central | Lop Buri | 19.1 (18.7, 19.5) | 16.4 (16.1, 16.8) |
| Central | Phra Nakhon Sri Ayuthaya | 20.1 (19.6, 20.6) | 17.0 (16.7, 17.4) |
| Central | Saraburi | 19.2 (18.8, 19.7) | 16.5 (16.2, 16.9) |
| Central | Singburi | 19.8 (19.2, 20.5) | 16.9 (16.3, 17.5) |
| East | Chachoengsao | 20.3 (19.9, 20.8) | 17.2 (16.8, 17.6) |
| East | Chanthaburi | 20.2 (19.7, 20.8) | 17.4 (17.0, 17.8) |
| East | Chon Buri | 20.7 (20.3, 21.1) | 18.1 (17.8, 18.5) |
| East | Nakhon Nayok | 20.0 (19.4, 20.6) | 17.1 (16.5, 17.6) |
| East | Prachin Buri | 20.1 (19.6, 20.7) | 16.6 (16.2, 17.0) |
| East | Rayong | 20.6 (20.1, 21.2) | 17.5 (17.1, 18.0) |
| East | Sa Kaew | 18.9 (18.5, 19.4) | 15.7 (15.4, 16.1) |
| East | Trat | 20.5 (19.8, 21.3) | 17.2 (16.6, 17.8) |
| North | Chiang Mai | 19.5 (19.2, 19.8) | 16.6 (16.3, 16.8) |
| North | Chiang Rai | 19.3 (19.0, 19.6) | 16.8 (16.5, 17.1) |
| North | Kam Phaeng Phet | 18.4 (18.1, 18.8) | 16.1 (15.8, 16.4) |
| North | Lampang | 18.9 (18.6, 19.3) | 16.5 (16.2, 16.8) |
| North | Lamphun | 19.1 (18.7, 19.6) | 16.2 (15.9, 16.6) |
| North | Mae Hong Son | 19.9 (19.2, 20.6) | 17.8 (17.2, 18.4) |
| North | Nakhon Sawan | 18.9 (18.6, 19.2) | 16.3 (16.0, 16.6) |
| North | Nan | 19.9 (19.5, 20.4) | 17.9 (17.4, 18.3) |
| North | Phayao | 18.3 (17.9, 18.7) | 15.9 (15.6, 16.3) |
| North | Phetchabun | 18.6 (18.3, 19.0) | 15.9 (15.6, 16.2) |
| North | Phichit | 19.1 (18.7, 19.5) | 16.4 (16.1, 16.8) |
| North | Phitsanulok | 19.0 (18.7, 19.4) | 16.9 (16.6, 17.3) |
| North | Phrae | 18.8 (18.4, 19.3) | 15.4 (15.1, 15.8) |
| North | Sukhothai | 18.7 (18.3, 19.1) | 16.3 (15.9, 16.6) |
| North | Tak | 18.7 (18.3, 19.2) | 16.8 (16.4, 17.2) |
| North | Uthai Thani | 19.0 (18.5, 19.5) | 16.6 (16.2, 17.1) |
| North | Uttaradit | 18.8 (18.4, 19.2) | 16.2 (15.9, 16.6) |
| Northeast | Amnat Chareon | 19.6 (19.1, 20.2) | 16.5 (16.1, 16.9) |
| Northeast | Bueng Kan | 18.8 (18.3, 19.3) | 16.3 (15.9, 16.7) |
| Northeast | Buri Ram | 19.2 (19.0, 19.6) | 16.6 (16.3, 16.8) |
| Northeast | Chaiyaphum | 18.3 (18.1, 18.6) | 16.0 (15.7, 16.3) |
| Northeast | Kalasin | 18.7 (18.4, 19.0) | 15.7 (15.4, 16.0) |
| Northeast | Khon Kaen | 19.1 (18.8, 19.4) | 16.2 (16.0, 16.4) |
| Northeast | Loei | 19.1 (18.7, 19.5) | 16.7 (16.4, 17.1) |
| Northeast | Mukdahan | 18.8 (18.3, 19.4) | 17.1 (16.6, 17.6) |
| Northeast | Naha Sarakham | 18.4 (18.1, 18.8) | 15.6 (15.3, 15.9) |
| Northeast | Nakhon Phanom | 18.1 (17.8, 18.5) | 15.6 (15.3, 15.9) |
| Northeast | Nakhon Ratchasima | 19.6 (19.3, 19.9) | 16.9 (16.7, 17.1) |
| Northeast | Nong Khai | 18.9 (18.4, 19.3) | 16.6 (16.2, 17.0) |
| Northeast | Nongbua Lamphu | 18.6 (18.2, 19.1) | 16.4 (16.0, 16.8) |
| Northeast | Roi Et | 19.0 (18.7, 19.3) | 15.9 (15.7, 16.2) |
| Northeast | Sakon Nakhon | 18.7 (18.3, 19.0) | 16.1 (15.8, 16.4) |
| Northeast | Si Sa Ket | 19.1 (18.9, 19.4) | 16.6 (16.4, 16.9) |
| Northeast | Surin | 19.4 (19.1, 19.7) | 16.9 (16.6, 17.2) |
| Northeast | Ubon Ratchathani | 19.1 (18.9, 19.4) | 16.9 (16.7, 17.2) |
| Northeast | Udon Thani | 18.6 (18.3, 18.9) | 15.9 (15.7, 16.1) |
| Northeast | Yasothon | 18.4 (18.0, 18.8) | 15.7 (15.4, 16.1) |
| South | Chumphon | 21.1 (20.6, 21.7) | 17.5 (17.1, 18.0) |
| South | Krabi | 21.2 (20.6, 21.9) | 17.7 (17.2, 18.2) |
| South | Nakhon Si Thammarat | 21.4 (21.0, 21.8) | 17.7 (17.3, 18.0) |
| South | Narathiwat | 18.8 (18.4, 19.3) | 16.7 (16.3, 17.1) |
| South | Pattani | 19.2 (18.8, 19.7) | 16.4 (16.0, 16.8) |
| South | Phangnga | 20.3 (19.7, 21.1) | 18.3 (17.7, 19.0) |
| South | Phatthalung | 22.4 (21.8, 23.0) | 18.0 (17.6, 18.5) |
| South | Phuket | 20.8 (20.2, 21.5) | 18.0 (17.5, 18.6) |
| South | Ranong | 20.9 (20.1, 21.8) | 18.2 (17.5, 19.0) |
| South | Satun | 20.6 (19.9, 21.3) | 18.1 (17.6, 18.8) |
| South | Songkhla | 21.3 (21.0, 21.8) | 17.5 (17.2, 17.8) |
| South | Surat Thani | 21.7 (21.3, 22.2) | 18.0 (17.7, 18.4) |
| South | Trang | 21.5 (21.0, 22.1) | 17.9 (17.5, 18.4) |
| South | Yala | 20.2 (19.7, 20.8) | 17.4 (16.9, 17.9) |
| West | Kanchanaburi | 19.6 (19.2, 20.1) | 16.9 (16.6, 17.3) |
| West | Phachuap Khiri Khan | 21.0 (20.5, 21.6) | 17.8 (17.3, 18.2) |
| West | Phetchaburi | 20.7 (20.2, 21.3) | 17.6 (17.2, 18.1) |
| West | Ratchaburi | 20.3 (19.9, 20.8) | 16.6 (16.3, 17.0) |
| West | Samut Songkhram | 20.8 (20.1, 21.5) | 17.2 (16.6, 17.9) |
| West | Suphan Buri | 19.4 (19.0, 19.8) | 16.3 (16.0, 16.7) |
