# Supplementary material for: Survival and life expectancy inequality by gender in Thai provinces: Trends from 2015 to 2023
Source: PLoS One. 2026 May 13;21(5):e0348587. doi: 10.1371/journal.pone.0348587 (PMC13170844; doi:10.1371/journal.pone.0348587)
Supplement: S3 Table — Values shown as mean (lower, upper); bounds are 95% interval limits derived from mortality uncertainty. (DOCX) [file pone.0348587.s004.docx]

**S3 Table. Probability of surviving from age 20 to 65 (**${}_{\boldsymbol{20}}{\boldsymbol{p}_{\boldsymbol{65}}}$ **) by province and sex, Thailand, 2023. Values shown as mean (lower, upper); bounds are 95% interval limits derived from mortality uncertainty.**

| **Region** | **Province** | **Female** | **Male** |
| --- | --- | --- | --- |
| **Thailand** | | **0.882 (0.877, 0.887)** | **0.739 (0.731, 0.747)** |
| Bangkok | Bangkok | 0.913 (0.911, 0.916) | 0.804 (0.800, 0.808) |
| Peripheral area | Nakhon Pathom | 0.893 (0.888, 0.899) | 0.765 (0.757, 0.773) |
| Peripheral area | Nonthaburi | 0.908 (0.904, 0.912) | 0.787 (0.780, 0.793) |
| Peripheral area | Pathum Thani | 0.893 (0.888, 0.898) | 0.781 (0.773, 0.788) |
| Peripheral area | Samut Prakan | 0.900 (0.895, 0.904) | 0.777 (0.770, 0.784) |
| Peripheral area | Samut Sakhon | 0.901 (0.895, 0.907) | 0.778 (0.768, 0.788) |
| Central | Ang Thong | 0.864 (0.855, 0.873) | 0.708 (0.694, 0.722) |
| Central | Chai Nat | 0.861 (0.852, 0.869) | 0.697 (0.684, 0.710) |
| Central | Lop Buri | 0.857 (0.850, 0.864) | 0.705 (0.696, 0.715) |
| Central | Phra Nakhon Sri Ayuthaya | 0.873 (0.867, 0.879) | 0.723 (0.714, 0.732) |
| Central | Saraburi | 0.860 (0.853, 0.867) | 0.709 (0.699, 0.720) |
| Central | Singburi | 0.869 (0.859, 0.879) | 0.719 (0.704, 0.735) |
| East | Chachoengsao | 0.876 (0.870, 0.882) | 0.738 (0.729, 0.747) |
| East | Chanthaburi | 0.874 (0.867, 0.881) | 0.742 (0.732, 0.752) |
| East | Chon Buri | 0.882 (0.877, 0.886) | 0.761 (0.754, 0.768) |
| East | Nakhon Nayok | 0.871 (0.862, 0.880) | 0.734 (0.720, 0.748) |
| East | Prachin Buri | 0.873 (0.865, 0.880) | 0.721 (0.710, 0.732) |
| East | Rayong | 0.880 (0.874, 0.887) | 0.746 (0.737, 0.756) |
| East | Sa Kaew | 0.853 (0.845, 0.861) | 0.696 (0.685, 0.706) |
| East | Trat | 0.879 (0.869, 0.888) | 0.737 (0.723, 0.752) |
| North | Chiang Mai | 0.882 (0.878, 0.885) | 0.721 (0.715, 0.728) |
| North | Chiang Rai | 0.878 (0.874, 0.883) | 0.728 (0.721, 0.735) |
| North | Kam Phaeng Phet | 0.865 (0.859, 0.871) | 0.707 (0.698, 0.716) |
| North | Lampang | 0.873 (0.868, 0.879) | 0.720 (0.711, 0.728) |
| North | Lamphun | 0.876 (0.869, 0.882) | 0.711 (0.700, 0.722) |
| North | Mae Hong Son | 0.887 (0.878, 0.897) | 0.755 (0.741, 0.769) |
| North | Nakhon Sawan | 0.872 (0.868, 0.877) | 0.713 (0.705, 0.720) |
| North | Nan | 0.888 (0.882, 0.894) | 0.757 (0.747, 0.767) |
| North | Phayao | 0.863 (0.856, 0.869) | 0.701 (0.691, 0.712) |
| North | Phetchabun | 0.869 (0.863, 0.874) | 0.701 (0.693, 0.709) |
| North | Phichit | 0.875 (0.869, 0.881) | 0.718 (0.708, 0.728) |
| North | Phitsanulok | 0.875 (0.870, 0.880) | 0.732 (0.724, 0.740) |
| North | Phrae | 0.872 (0.865, 0.878) | 0.686 (0.675, 0.697) |
| North | Sukhothai | 0.870 (0.864, 0.875) | 0.712 (0.703, 0.722) |
| North | Tak | 0.870 (0.863, 0.877) | 0.729 (0.719, 0.739) |
| North | Uthai Thani | 0.875 (0.867, 0.882) | 0.723 (0.711, 0.736) |
| North | Uttaradit | 0.871 (0.864, 0.877) | 0.711 (0.701, 0.722) |
| Northeast | Amnat Chareon | 0.886 (0.879, 0.893) | 0.722 (0.710, 0.734) |
| Northeast | Bueng Kan | 0.873 (0.866, 0.881) | 0.716 (0.704, 0.728) |
| Northeast | Buri Ram | 0.881 (0.877, 0.885) | 0.725 (0.718, 0.731) |
| Northeast | Chaiyaphum | 0.867 (0.862, 0.871) | 0.707 (0.700, 0.715) |
| Northeast | Kalasin | 0.872 (0.867, 0.877) | 0.699 (0.691, 0.707) |
| Northeast | Khon Kaen | 0.878 (0.875, 0.882) | 0.714 (0.708, 0.720) |
| Northeast | Loei | 0.878 (0.873, 0.884) | 0.728 (0.719, 0.737) |
| Northeast | Mukdahan | 0.874 (0.866, 0.882) | 0.740 (0.728, 0.752) |
| Northeast | Naha Sarakham | 0.868 (0.863, 0.873) | 0.695 (0.687, 0.703) |
| Northeast | Nakhon Phanom | 0.863 (0.857, 0.869) | 0.695 (0.685, 0.704) |
| Northeast | Nakhon Ratchasima | 0.886 (0.882, 0.889) | 0.734 (0.729, 0.739) |
| Northeast | Nong Khai | 0.875 (0.868, 0.881) | 0.726 (0.716, 0.736) |
| Northeast | Nongbua Lamphu | 0.871 (0.865, 0.878) | 0.718 (0.708, 0.729) |
| Northeast | Roi Et | 0.877 (0.873, 0.881) | 0.706 (0.699, 0.713) |
| Northeast | Sakon Nakhon | 0.872 (0.867, 0.876) | 0.711 (0.703, 0.719) |
| Northeast | Si Sa Ket | 0.879 (0.875, 0.883) | 0.727 (0.720, 0.734) |
| Northeast | Surin | 0.883 (0.879, 0.887) | 0.733 (0.727, 0.740) |
| Northeast | Ubon Ratchathani | 0.879 (0.875, 0.883) | 0.735 (0.729, 0.741) |
| Northeast | Udon Thani | 0.871 (0.867, 0.875) | 0.705 (0.698, 0.711) |
| Northeast | Yasothon | 0.868 (0.861, 0.874) | 0.699 (0.689, 0.709) |
| South | Chumphon | 0.891 (0.884, 0.897) | 0.771 (0.761, 0.780) |
| South | Krabi | 0.893 (0.885, 0.900) | 0.774 (0.763, 0.785) |
| South | Nakhon Si Thammarat | 0.894 (0.890, 0.898) | 0.773 (0.767, 0.779) |
| South | Narathiwat | 0.856 (0.849, 0.863) | 0.748 (0.739, 0.758) |
| South | Pattani | 0.863 (0.856, 0.870) | 0.741 (0.731, 0.752) |
| South | Phangnga | 0.880 (0.871, 0.889) | 0.788 (0.775, 0.801) |
| South | Phatthalung | 0.907 (0.901, 0.912) | 0.781 (0.772, 0.791) |
| South | Phuket | 0.887 (0.879, 0.895) | 0.782 (0.770, 0.794) |
| South | Ranong | 0.888 (0.877, 0.899) | 0.787 (0.771, 0.802) |
| South | Satun | 0.884 (0.875, 0.892) | 0.785 (0.772, 0.797) |
| South | Songkhla | 0.894 (0.890, 0.898) | 0.769 (0.762, 0.776) |
| South | Surat Thani | 0.898 (0.894, 0.903) | 0.782 (0.774, 0.789) |
| South | Trang | 0.896 (0.890, 0.902) | 0.778 (0.769, 0.788) |
| South | Yala | 0.878 (0.871, 0.886) | 0.767 (0.756, 0.778) |
| West | Kanchanaburi | 0.868 (0.862, 0.875) | 0.742 (0.733, 0.750) |
| West | Phachuap Khiri Khan | 0.889 (0.882, 0.896) | 0.763 (0.753, 0.773) |
| West | Phetchaburi | 0.885 (0.878, 0.891) | 0.760 (0.750, 0.770) |
| West | Ratchaburi | 0.880 (0.874, 0.885) | 0.734 (0.725, 0.742) |
| West | Samut Songkhram | 0.886 (0.877, 0.895) | 0.749 (0.734, 0.765) |
| West | Suphan Buri | 0.865 (0.859, 0.871) | 0.724 (0.716, 0.733) |
